# Supplementary material for: Modeling glioblastoma heterogeneity as a dynamic network of cell states
Source: Mol Syst Biol. 2021 Sep 16;17(9):e10105. doi: 10.15252/msb.202010105 (PMC8444284; doi:10.15252/msb.202010105)
Supplement: Supplementary file 6 — Source Data for Figure 5 [file MSB-17-e10105-s004.zip › Figure5A_sourcedata/GSEA_3071/nosigdb_stateV.GseaPreranked.1622795159954/IVY_CTMVP.html]

Details for gene set IVY\_CTMVP[GSEA]

|  || Dataset | state43071 |
| Phenotype | NoPhenotypeAvailable |
| Upregulated in class | na\_neg |
| GeneSet | IVY\_CTMVP |
| Enrichment Score (ES) | -0.7125298 |
| Normalized Enrichment Score (NES) | -3.821733 |
| Nominal p-value | 0.0 |
| FDR q-value | 0.0 |
| FWER p-Value | 0.0 |
Table: GSEA Results Summary

  

Fig 1: Enrichment plot: IVY\_CTMVP      
 Profile of the Running ES Score & Positions of GeneSet Members on the Rank Ordered List

  

| PROBE | GENE SYMBOL | GENE\_TITLE | RANK IN GENE LIST | RANK METRIC SCORE | RUNNING ES | CORE ENRICHMENT || 1 | PDGFB |  |  | 283 | 1.080 | -0.0438 | No |
| 2 | INPP4B |  |  | 319 | 1.047 | -0.0403 | No |
| 3 | MCAM |  |  | 370 | 0.980 | -0.0403 | No |
| 4 | APOLD1 |  |  | 372 | 0.980 | -0.0309 | No |
| 5 | STC1 |  |  | 433 | 0.920 | -0.0334 | No |
| 6 | A2M |  |  | 532 | 0.846 | -0.0440 | No |
| 7 | CCDC102B |  |  | 551 | 0.830 | -0.0393 | No |
| 8 | HAPLN1 |  |  | 582 | 0.810 | -0.0372 | No |
| 9 | COL1A2 |  |  | 1028 | 0.614 | -0.1166 | No |
| 10 | KCNJ2 |  |  | 1033 | 0.613 | -0.1113 | No |
| 11 | GNG11 |  |  | 1219 | 0.556 | -0.1414 | No |
| 12 | PLXDC1 |  |  | 1487 | 0.483 | -0.1879 | No |
| 13 | RAMP2 |  |  | 1559 | 0.468 | -0.1970 | No |
| 14 | FRZB |  |  | 1884 | 0.406 | -0.2552 | No |
| 15 | CDH6 |  |  | 2101 | 0.374 | -0.2930 | No |
| 16 | ACTA2 |  |  | 2227 | 0.359 | -0.3134 | No |
| 17 | NID1 |  |  | 2297 | 0.350 | -0.3233 | No |
| 18 | ECM1 |  |  | 2564 | 0.315 | -0.3712 | No |
| 19 | PRDM1 |  |  | 2921 | 0.277 | -0.4368 | No |
| 20 | MYO1B |  |  | 3217 | -0.254 | -0.4910 | No |
| 21 | COL18A1 |  |  | 3251 | -0.259 | -0.4948 | No |
| 22 | CRIP1 |  |  | 3514 | -0.302 | -0.5421 | No |
| 23 | ITGB1 |  |  | 3614 | -0.317 | -0.5580 | No |
| 24 | SVIL |  |  | 3763 | -0.342 | -0.5830 | No |
| 25 | FOXC1 |  |  | 3889 | -0.365 | -0.6035 | No |
| 26 | ISLR |  |  | 3936 | -0.375 | -0.6086 | No |
| 27 | FOXF2 |  |  | 4055 | -0.400 | -0.6274 | No |
| 28 | LAMB1 |  |  | 4115 | -0.413 | -0.6346 | No |
| 29 | PXDN |  |  | 4176 | -0.430 | -0.6420 | No |
| 30 | PLAC9 |  |  | 4197 | -0.437 | -0.6415 | No |
| 31 | PHACTR2 |  |  | 4213 | -0.444 | -0.6401 | No |
| 32 | FSTL1 |  |  | 4262 | -0.460 | -0.6448 | No |
| 33 | PDE5A |  |  | 4271 | -0.465 | -0.6418 | No |
| 34 | F2R |  |  | 4316 | -0.476 | -0.6456 | No |
| 35 | EDNRA |  |  | 4361 | -0.489 | -0.6492 | No |
| 36 | UACA |  |  | 4621 | -0.604 | -0.6930 | No |
| 37 | IGFBP7 |  |  | 4622 | -0.604 | -0.6871 | No |
| 38 | S1PR3 |  |  | 4625 | -0.605 | -0.6816 | No |
| 39 | ARHGAP18 |  |  | 4693 | -0.642 | -0.6882 | No |
| 40 | ITGA1 |  |  | 4706 | -0.647 | -0.6842 | No |
| 41 | PCDH18 |  |  | 4803 | -0.717 | -0.6956 | No |
| 42 | EBF1 |  |  | 4831 | -0.738 | -0.6936 | No |
| 43 | NR2F2 |  |  | 4917 | -0.830 | -0.7018 | No |
| 44 | KDELR3 |  |  | 4974 | -0.893 | -0.7038 | Yes |
| 45 | PAPSS2 |  |  | 4983 | -0.901 | -0.6966 | Yes |
| 46 | PRKG1 |  |  | 5028 | -0.988 | -0.6953 | Yes |
| 47 | SERPINH1 |  |  | 5059 | -1.050 | -0.6909 | Yes |
| 48 | CTGF |  |  | 5070 | -1.073 | -0.6823 | Yes |
| 49 | PROCR |  |  | 5097 | -1.139 | -0.6762 | Yes |
| 50 | FILIP1L |  |  | 5103 | -1.158 | -0.6658 | Yes |
| 51 | COL5A2 |  |  | 5112 | -1.189 | -0.6557 | Yes |
| 52 | PDLIM1 |  |  | 5114 | -1.191 | -0.6443 | Yes |
| 53 | TGFBI |  |  | 5136 | -1.259 | -0.6360 | Yes |
| 54 | OLFML2A |  |  | 5140 | -1.270 | -0.6242 | Yes |
| 55 | LBH |  |  | 5153 | -1.323 | -0.6136 | Yes |
| 56 | GPX8 |  |  | 5171 | -1.418 | -0.6030 | Yes |
| 57 | CD248 |  |  | 5178 | -1.448 | -0.5900 | Yes |
| 58 | PRSS23 |  |  | 5189 | -1.489 | -0.5774 | Yes |
| 59 | THBS1 |  |  | 5197 | -1.538 | -0.5637 | Yes |
| 60 | COL4A2 |  |  | 5198 | -1.545 | -0.5487 | Yes |
| 61 | MFAP2 |  |  | 5208 | -1.592 | -0.5348 | Yes |
| 62 | BGN |  |  | 5216 | -1.654 | -0.5200 | Yes |
| 63 | OLFML3 |  |  | 5224 | -1.700 | -0.5048 | Yes |
| 64 | IGFBP4 |  |  | 5226 | -1.725 | -0.4881 | Yes |
| 65 | DCN |  |  | 5233 | -1.798 | -0.4717 | Yes |
| 66 | TM4SF1 |  |  | 5235 | -1.812 | -0.4542 | Yes |
| 67 | SNAI2 |  |  | 5241 | -1.903 | -0.4366 | Yes |
| 68 | TPM2 |  |  | 5247 | -1.967 | -0.4184 | Yes |
| 69 | PCOLCE |  |  | 5248 | -1.999 | -0.3988 | Yes |
| 70 | MGP |  |  | 5252 | -2.037 | -0.3795 | Yes |
| 71 | PDGFRB |  |  | 5253 | -2.045 | -0.3596 | Yes |
| 72 | CFH |  |  | 5257 | -2.141 | -0.3392 | Yes |
| 73 | COL4A1 |  |  | 5266 | -2.279 | -0.3185 | Yes |
| 74 | CTHRC1 |  |  | 5270 | -2.394 | -0.2957 | Yes |
| 75 | ACTG2 |  |  | 5276 | -2.506 | -0.2722 | Yes |
| 76 | LUM |  |  | 5277 | -2.507 | -0.2478 | Yes |
| 77 | ARHGAP29 |  |  | 5278 | -2.576 | -0.2226 | Yes |
| 78 | MYL9 |  |  | 5280 | -2.643 | -0.1970 | Yes |
| 79 | TAGLN |  |  | 5281 | -2.669 | -0.1709 | Yes |
| 80 | FN1 |  |  | 5287 | -2.866 | -0.1439 | Yes |
| 81 | FOXS1 |  |  | 5291 | -3.324 | -0.1120 | Yes |
| 82 | COL3A1 |  |  | 5292 | -3.630 | -0.0766 | Yes |
| 83 | COL6A3 |  |  | 5293 | -3.722 | -0.0403 | Yes |
| 84 | COL1A1 |  |  | 5294 | -4.144 | 0.0002 | Yes |
Table: GSEA details [plain text format]

  

Fig 2: IVY\_CTMVP: Random ES distribution      
 Gene set null distribution of ES for **IVY\_CTMVP**
